# Psychometric Properties of the Spanish Versions of the Activity Restriction Scale

**DOI:** 10.3390/geriatrics10010024

**Published:** 2025-02-10

**Authors:** Laura García-García, Lucía Jiménez-Gonzalo, José A. Fernandes-Pires, Brent T. Mausbach, Luis Manuel Pérez-Cardona, Claudia Benito-Rincón, Andrés Losada-Baltar

**Affiliations:** 1 Department of Psychology, Faculty of Health Sciences, Rey Juan Carlos University, 28922 Madrid, Spain; laura.ggarcia@urjc.es (L.G.-G.); lucia.jimenez@urjc.es (L.J.-G.); jose.fernandes@urjc.es (J.A.F.-P.); lm.perez.2018@alumnos.urjc.es (L.M.P.-C.); c.benitor.2018@alumnos.urjc.es (C.B.-R.); 2 Department of Psychiatry, University of California San Diego, San Diego, CA 92093, USA; bmausbach@health.ucsd.edu

**Keywords:** activity restriction, anxiety, depression, family caregivers, old adults, perceived health

## Abstract

**Introduction:** Activity restriction (i.e., feeling limited in performing certain activities) has been studied in relation to psychological distress. The Activity Restriction Scale (ARS) has been widely used, with a two-factor structure obtained in previous studies. However, there is no validated instrument for this measure in Spanish. This study aims to analyze the psychometric properties of the Spanish version of the original ARS and to create an adapted version for older adults. **Method:** In Phase 1, the original ARS was tested in 143 Spanish family caregivers. In Phase 2, this version was explored through a pilot study with 10 Spanish older adults. In Phase 3, the ARS-Older Adults version (ARS-OA) was tested in a sample of 246 Spanish older adults. Confirmatory factor analysis was conducted in Phases 1 and 3, in addition to testing reliability, and convergent and concurrent validity. A descriptive analysis was carried out in Phase 2. **Results:** Both factor analyses provided support for a two-factor solution: instrumental and expressive activity restriction. Cronbach’s alpha was 0.86 (Phase 1) and 0.81 (Phase 3). The data also suggested good convergent and concurrent validity. **Conclusions:** The results revealed good psychometric properties of both versions of the ARS, suggesting that it is a suitable instrument for assessing activity restriction in Spanish-speaking populations. Activity restriction is suggested as a relevant variable to take into account in understanding people’s mental health.

## 1. Introduction

Depression is a relevant topic in general and also in the field of gerontology as it is associated with decreases in the quality of life and increases in frailty and disability in middle-aged and older adults [1]. It is also a key problem for family caregivers of older adults with health problems, such as dementia, as it has a negative influence on the quality of care provided and received [2]. Analyzing the factors that help explain depressive symptoms in these populations can provide relevant information for developing interventions aimed at improving the mental health of older adults and caregivers. One of these factors is activity restriction. According to the Activity Restriction Model of Depressed Affect [3,4], activity restriction can be defined as the interferences in normal social and recreational activities that may occur as a result of life stresses, and may contribute to increases in depressive symptoms and other mental health problems. In fact, activity restriction has been found to be cross-sectionally and longitudinally associated with mental and physical health problems [5,6,7,8,9,10], both directly or indirectly, for example, exacerbating the effects of stress on people’s health [11]. Activity restriction has also been found to be related to mental and physical health in older adults. For example, there are studies showing the mediating role of activity restriction in the association between pain [4,12,13] and functional limitation [14] with depressive symptoms in older age. Longitudinal data link higher levels of activity restriction with late-life depression [15].

Activity restriction has been measured using different procedures, from single-item measures [16,17] to scales such as the Groningen Activity Restriction Scale for measuring disability (GARS; [18]) or the Activity Restriction Scale (ARS; [4,12,19]), which are the most widely used instruments. The GARS is a measure specifically designed to assess disability in activities of daily and instrumental activities. In agreement with Mausbach et al. [20], this study is particularly interested in the analysis of restriction to social and recreational activities as a function of life situations that can be considered stressful or challenging, such as providing care for a relative with a chronic health problem, or coping with the aging process. For this reason, the study will focus on the ARS, a scale developed for measuring the extent to which people with pain or disability felt restricted when engaging in activities such as self-care, doing household chores, hobbies, or maintaining friendships [4,12,19].

The ARS originally requested that study participants indicate the extent to which nine areas of activity in their lives were restricted, including going to work. However, other studies, such as those conducted by Lee et al. [5] or Loucks-Atkinson et al. [21], used an eight-item version of the ARS, excluding the item “going to work” in the case of the Loucks-Atkinson et al. study [21]. Even though the ARS has been widely used, studies analyzing its psychometric properties are lacking. To our knowledge, only two studies, by Loucks-Atkinson [21,22], make reference to psychometric properties of the ARS beyond internal consistency. Using the eight-item version of the scale, Loucks-Atkinson [22] found support for a two-factor structure. The first factor was labeled “instrumental activity restriction” as it included activities that were considered necessary and instrumental (caring for yourself, caring for others, doing household chores, and going shopping), while the second factor was labeled “expressive activity restriction” as it included activities that were considered less essential and more intrinsically motivated (sports and recreation, visiting friends, working on hobbies, and maintaining friendship) [22]. The factors showed good reliability indices (0.79 and 0.84, respectively) and significant associations with more depressive symptoms and lower social support and physical health [21].

As no study has analyzed the psychometric properties of a Spanish version of the ARS [4,12,19], and given the relevance of this construct in old age, the objectives of this study were to validate the Spanish version of the original scale and to adapt this scale in order to create a version for use with older adults. For this purpose, three phases were carried out: (1) to study the psychometric properties of the original nine-item ARS [12] in a sample of Spanish family caregivers of people with dementia; (2) to conduct a pilot study using this original scale in a sample of Spanish community-dwelling older adults; and (3) to test the psychometric properties of an adaptation of this scale for older adults, excluding the item “going to work” and splitting the item “sports and recreation” in two (Activity Restriction Scale-Older Adults version; ARS-OA).

## 2. Phase 1: Spanish Validation of the Original Activity Restriction Scale (ARS)

### 2.1. Method

#### 2.1.1. Participants and Procedure

The participants were 143 Spanish dementia family caregivers. The descriptive data of the sample are shown in the Results section in Table 1. The inclusion criteria were: (1) being over 18 years old; (2) self-identifying as the primary caregiver of a family member with dementia; and (3) spending more than one hour per day on caregiving duties.

The project that allowed the sample recruitment was approved by the Ethics Committee of the Rey Juan Carlos University (internal registration number: 23032012022012) in April 2012. All participants were recruited through collaborating day-care centers, all of which belong to the Community of Madrid. Data collection was carried out in person through face-to-face interviews between January 2013 and December 2015. All the participants completed the informed consent before beginning the evaluation.

#### 2.1.2. Measures

Sociodemographic variables: the age, gender, marital status, educational level, and employment status of the participants were collected.

Activity restriction. Activity restriction was measured through the Activity Restriction Scale (ARS; [12]). Participants were asked to rate the extent to which they perceived restriction in nine areas of activity (e.g., “caring for self”; Table 2). Answers ranged from 0 (“never or seldom restricted”) to 4 (“greatly restricted”). The psychometric properties obtained for the ARS in this study are shown in the Results section. The final version of the ARS, in both English and Spanish (“Escala de Restricción Conductual”), with the corresponding scoring form, can be found in Supplementary Material.

Engagement in leisure activities. This was assessed through a Spanish adaptation [23] of the Leisure Time Satisfaction Scale (LTS; [24]), a questionnaire created to evaluate engagement in leisure activities or behavioral activation by family caregivers. It consists of six items (e.g., “taking part in hobbies or other interests”). The response options were “not at all”, “a little”, and “frequently”. Higher scores indicate more engagement in leisure activities. In this study, Cronbach’s α was 0.67.

Depressive symptoms. The Spanish version [25] of the Center for Epidemiological Studies Scale (CES-D; [26]) was used. It has 20 items (e.g., “I felt sad”), where the response options consisted of a 4-point Likert scale with a range from 0 (“rarely or none of the time”) to 3 (“most or all of the time”). Higher scores indicate a greater frequency of depressive symptoms. Cronbach’s α in this study was 0.89.

Anxiety symptoms. These were assessed using the Spanish version [27] of the tension–anxiety subscale from the Profile of Mood States (POMS; [28]). It consists of nine items (e.g., “how often have you felt tense”). Responses are rated on a Likert-type scale ranging from 0 (“not at all”) to 4 (“extremely”). Higher scores indicate more anxious symptoms. Cronbach’s α was 0.89.

Perceived health. This was assessed through the item “In general, would you say your health is”, with Likert-type response options ranging from 0 (“very bad”) to 4 (“very good”). Higher scores on this item reflect better perceived health. According to Macias et al. [29], this question is an appropriate measure for assessing physical health.

#### 2.1.3. Data Analysis

Sample, items, and scale characteristics were analyzed through means, standard deviations, percentages, and ranges, among other descriptive analysis.

A confirmatory factor analysis (CFA) was conducted to test whether the Spanish version of the ARS adjusts well to the two-factor structure obtained in previous studies [21,22]. It was performed using the weighted least squares mean and variances adjusted (WLSMV) estimation method, which is appropriate for ordinal variables [30]. To check the fit of the model to the data, we used: chi-square test (χ^2^), root mean square error of approximation (RMSEA) and its 90% confidence interval, weighted root mean square residual (WRMR), comparative fit index (CFI) and the Tucker–Lewis index (TLI). Considering Hu and Bentler [31], the following cutoff points were used for the fit indices: RMSEA close to 0.06 and CFI/TLI close to 0.95. According to DiStefano et al. [32], WRMR values below 1.0 indicate an acceptable fit. Considering Bollen [33], if dividing the χ^2^ value by its degrees of freedom (χ^2^/df) gives a score of less than 3, the model is considered to be a good fit.

Reliability was measured through internal consistency using Cronbach’s alpha for the entire scale as well as for each individual factor. Item-test correlations were calculated using Pearson correlations. Convergent validity was studied through Pearson correlations between the ARS and the measure of engagement in leisure activities. Concurrent validity was found by Pearson correlations between the ARS and symptoms of depression and anxiety and perceived health. The CFA were calculated using Mplus 7.0 software [34]. IBM SPSS Statistics 28 was used to calculate descriptive statistics, reliability coefficients, and Pearson correlations.

### 2.2. Results

#### 2.2.1. Descriptive Data of the Sample

Table 1 shows the characteristics of the sample.

#### 2.2.2. Confirmatory Factor Analysis (CFA)

The fit indices showed that the ARS has a good fit with the two-factor structure: χ^2^ (26) = 45.39, *p* < 0.05; χ^2^/df = 1.75; RMSEA = 0.07 (90% CI = 0.03–0.11); WRMR = 0.57; CFI = 0.99; TLI = 0.99. The first factor corresponds to the original instrumental activity restriction factor, and the second factor corresponds to the original expressive activity restriction factor. Both factors correlated with each other (r = 0.58; *p* < 0.01) and with the total scale (first factor: r = 0.85; *p* < 0.01; second factor: r = 0.92; *p* < 0.01) in a significant way. The factor loadings and descriptive statistics of the ARS are shown in Table 2 (all factor loadings were greater than 0.50.).

#### 2.2.3. Reliability

Cronbach’s α of the ARS was 0.86. For the first factor (instrumental activity restriction), Cronbach’s α was 0.77. For the second factor (expressive activity restriction), Cronbach’s α was 0.84. As can be seen in Table 2, all item-test correlations were significant, and the deletion of items did not contribute to a significant increase in Cronbach’s alpha value of the ARS.

#### 2.2.4. Convergent and Concurrent Validity

The associations between the assessed variables are shown in Table 3. Regarding convergent validity, the ARS and its factors correlated negatively and significantly with the engagement in leisure activities measure. With respect to concurrent validity, the full scale of the ARS and its factors correlated positively and significantly with symptoms of depression and anxiety. No significant association was found with perceived health.

## 3. Phase 2: Pilot Study of the Original ARS in Older Adults

In this phase, a pilot study was conducted with 10 community-dwelling older adults, with an age range of 65 to 80 years old (M = 70.70; SD = 4.40; 60% women). The inclusion criteria were: (1) being over 65 years old; (2) not having any sensorial or cognitive impairment that would prevent them from understanding the questions; and (3) not currently attending any day-care center. They were evaluated using the version of the ARS employed in Phase 1 [1] to test the feasibility of its use in this population. After analyzing the employment status of the sample, it was found that all the participants (100%) were retired, so it was considered appropriate to remove the item “going to work”. In addition, participants reported problems in understanding the item “participating in sports and recreation”, because they did not know which of the two constructs to answer, so it was decided that this item would be split into two: “participating in sports” and “participating in recreation activities”. The original developer of the scale (Richard Schulz) was contacted and gave his consent to make this adaptation of the scale for older adults. A translation and back-translation process was carried out following the recommendations of the International Test Commission (ITC) [35]. Finally, after this process, the Activity Restriction Scale-Older Adults version (ARS-OA; Table 4), which contained nine items, was obtained. The response options were the same as in the ARS (Phase 1). The final version of the ARS-OA, in both English and Spanish, is shown in Supplementary Material, with its corresponding score.

## 4. Phase 3: Older Adults’ Version of the Activity Restriction Scale (ARS-OA)

### 4.1. Method

#### 4.1.1. Participants and Procedure

The sample of this study was composed of 246 Spanish community-dwelling older adults. The characteristics of the sample are shown in Table 1. As can be seen, none of the participants were employed at the time of assessment. The inclusion criteria were: (1) being over 65 years old; (2) not having any sensorial or cognitive impairment that would prevent them from understanding the questions; and (3) not currently attending any day-care center.

This study was approved by the Ethics Committee of the Rey Juan Carlos University in June 2023 (internal registration number: 2802202310423). Participants were recruited from various centers, including cultural centers and senior centers, all located within the Community of Madrid and Guadalajara. Data collection was carried out from September 2023 to November 2024 and was conducted in person through interviews, and all the participants provided consent prior to the evaluation.

#### 4.1.2. Measures

Sociodemographic variables: participants’ age, gender, marital status, educational level, and employment status were assessed.

Activity restriction: the version of the ARS for older people obtained in Phase 2 was used in this study (Activity Restriction Scale-Older Adults version; ARS-OA; Table 4). It consists of nine items and the response options were the same as in the ARS (Phase 1): answers ranged from 0 (“never or seldom restricted”) to 4 (“greatly restricted”). The psychometric properties of the ARS-OA can be found in the Results section. The final version of the ARS-OA (in Spanish: “Escala de Restricción Conductual para Personas Mayores”) is available in Supplementary Material.

Behavioral activation: the activation subscale of the Spanish version [36] of the Behavioral Activation for Depression Scale-Short Form (BADS-SF; [37]) was used. It consists of five items (e.g., “I engaged in many different activities”), with response options ranging from 0 (“Not at all”) to 6 (“Completely”). Higher scores indicate greater behavioral activation. In this study, Cronbach’s α was 0.82.

Depressive symptoms: the same instrument described in Phase 1 was used. Cronbach’s alpha in this study was 0.84.

Anxiety symptoms: the Spanish version [38] of the Geriatric Anxiety Inventory (GAI; [39]) was used. This is a 20-item scale (e.g., “I spend a lot of time worrying”), where the response options were “Yes”/“No”. Higher scores indicate more symptoms of anxiety. In this study, Cronbach’s α was 0.90.

Perceived health: the same item was employed as in Phase 1.

#### 4.1.3. Data Analysis

The same data analysis was carried out as described in Phase 1.

### 4.2. Results

#### 4.2.1. Descriptive Data of the Sample

Descriptive characteristics of the sample are shown in Table 1.

#### 4.2.2. Confirmatory Factor Analysis (CFA)

The fit indices indicated that the ARS-OA demonstrated an excellent fit with the two-factor structure (instrumental activity restriction and expressive activity restriction): χ^2^ (26) = 43.21, *p* < 0.05; χ^2^/df = 1.66; RMSEA = 0.05 (90% CI = 0.02–0.07); WRMR = 0.65; CFI = 0.98; TLI = 0.98. The two factors were significantly correlated with each other (r = 0.61, *p* < 0.01), as well as with the total scale (first factor: r = 0.86, *p* < 0.01; second factor: r = 0.93, *p* < 0.01). Table 4 presents the factor loadings (all exceeded 0.50) and descriptive statistics.

#### 4.2.3. Reliability

The ARS-OA presented a Cronbach’s alpha of 0.81. Each of its factors had Cronbach’s alpha values of 0.68 (factor 1: instrumental activity restriction) and 0.72 (factor 2: expressive activity restriction). As shown in Table 4, all item-test correlations were significant, and no item contributed negatively to the total Cronbach’s alpha value.

#### 4.2.4. Convergent and Concurrent Validity

Table 3 shows the results obtained. Regarding convergent validity, there were negative and significant correlations between the measure of behavioral activation and the ARS-OA and its factors. Concerning concurrent validity, this version of the scale and its factors correlated positively and significantly with measures of depressive and anxious symptoms. In addition, the ARS-OA and its factors also correlated significantly with worse perceived health.

## 5. Discussion

The aims of this study were to provide preliminary evidence of the psychometric properties of the Spanish version of the Activity Restriction Scale (ARS; [12]) and of a version created for older adults (ARS-Older Adults version; ARS-OA). For this purpose, and considering previous studies [4,21,22], three phases were conducted. First, the original ARS [12] was tested in a sample of family caregivers of people with dementia. Second, this original version of the ARS was used in a pilot study with 10 older adults. Considering that most older adults are not engaged in paid-work activities, that the item “going to work” showed psychometric problems in previous studies, and that a better fit was obtained on excluding this item [21,22], the ARS-Older Adults version (ARS-OA) was developed by removing the item “going to work” and splitting the item “sports and recreation” in two. Third, the ARS-OA was analyzed in a sample of community-dwelling older adults.

On the one hand, the results obtained in Phase 1 suggest that the ARS shows psychometric properties that support its use with Spanish speakers. On the other hand, the psychometric properties of the ARS-OA found in Phase 3 suggest that it is a suitable instrument for assessing activity restriction in older adults. Specifically, the confirmatory factor analyses carried out for both phases support the two-factor structure previously proposed [21,22]. That is, this study provides support for the consideration of two dimensions of activity restriction: “instrumental activity restriction” and “expressive activity restriction”. In addition, both the original ARS and the ARS-OA show good reliability indices for the total scale, and for the individual factors, although in the case of the ARS-OA the reliability for the individual factors was lower, and could be considered acceptable. Regarding convergent and concurrent validity, and as expected, the ARS and the ARS-OA showed significant associations with indicators of behavioral activation and distress: higher scores in activity restriction were associated with lower engagement in leisure activities and lower behavioral activation, and higher levels of depressive and anxiety symptoms. However, while significant associations were found between activity restriction and lower subjective health in the sample of older adults (Phase 3), this association was not found in the caregiving sample (Phase 1). Future studies should further analyze this association and the potential reasons that might explain the observed findings, which might be potentially due to a higher prevalence of chronic health problems in the older adults sample.

Additional validity indicators have been obtained. For example, and as would be expected, higher mean scores in activity restriction have been observed in the sample of family caregivers of people with dementia (Phase 1) compared to the older adults’ sample (Phase 3). It is widely known that dementia caregivers face a large number of demands and stressors that are associated with high levels of distress (e.g., [2]). The obtained mean scores in depressive and anxiety symptomatology for caregivers are substantially higher than the usual cutoff scores suggesting clinical depression and anxiety [40], something not found for the older adults’ sample. Even though in this study activity restriction was analyzed in family caregivers of people with dementia, empirical support for the activity restriction model has also been obtained in other samples of caregivers, such as parental caregiving [41,42].

### 5.1. Limitations

This study has several limitations that should be considered. The sample size for each sub-study (Phases 1 and 3) could be considered small, and the cross-sectional design does not allow for testing predictive validity of the scales. In addition, both samples were composed of volunteer participants, and so the generalization of the results to the general population of caregivers or older adults may be compromised. As mentioned, although the reliability index obtained for the global ARS-OA scale can be seen as acceptable to good, the reliability obtained for the individual factors may be considered small.

### 5.2. Future Directions

Future studies should be carried out with larger sample sizes, analyzing both Spanish ARS and ARS-OA to provide support for the findings of this study. Longitudinal and random sampling studies should also be conducted to overcome the limitations discussed above. Moreover, future studies should further analyze the reliability of the ARS-OA and test if, for example, relevant areas for older adults are not tapped. This might be the case, for example, for feeling restricted in meeting the family, one of the most important valued areas for older adults, as shown by theoretical models such as the Socioemotional Selectivity Theory [43,44,45].

### 5.3. Practical Implications

The obtained findings seem to confirm that the variable activity restriction is relevant for understanding depression and anxiety. Therefore, the availability of these two scales for use with Spanish-speaking populations might contribute to further developments of strategies for understanding and treating mental health problems in Spanish populations.

### 5.4. Conclusions

This study is the first to provide psychometric support for the use of the Spanish version of the original ARS [12]. In addition, it offers an adaptation of the scale to be used with samples of older adults (ARS-OA). Both scales showed good psychometric properties, which suggest that they can be used for research and clinical purposes.

## Figures and Tables

**Table 1:** Descriptive data of the samples in Phase 1 and Phase 3.

		Mean (SD) or Percentage (N)/Range	
		Phase 1: Family Caregivers	Phase 3: Older Adults
Age		60.31 (13.20)/32–85	72.29 (5.62)/65–92
Gender	Male	22.4% (32)	22.4% (55)
	Female	77.6% (111)	77.6% (191)
Marital status	Married	61.5% (88)	49.2% (121)
	Widowed	2.8% (4)	27.6% (68)
	Single	30.1% (43)	5.7% (14)
	Divorced	3.5% (5)	10.2% (25)
	Separated	2.1% (3)	4.1% (10)
	Other (e.g., unmarried partner)	0% (0)	3.3% (8)
Educational level	No formal education	5.6% (8)	4.1% (10)
	Primary education	13.3% (19)	28.0% (69)
	Secondary education	7.7% (11)	18.3% (45)
	Completed high school	26.6% (38)	27.2% (67)
	University degree	37.8% (54)	20.7% (51)
	Postgraduate studies	8.4% (12)	1.6% (4)
Employment status	Employed	31.5% (45)	0% (0)
	Not employed	67.8% (97)	100% (246)

**Table 2:** Factor loadings and descriptive analysis of the Spanish Version of the ARS.

Original/Spanish Items	Factor 1 ^a^	Factor 2 ^b^	Item-Test r	Cronbach’s α Without the Item	Mean (SD)/Range
1. Caring for self/ Cuidado de sí mismo	0.70		0.64	0.86	1.81 (1.52)/0–4
2. Caring for others/ Cuidado de otros	0.71		0.65	0.86	1.45 (1.60)/0–4
3. Doing household chores/ Realizar tareas domésticas	0.73		0.62	0.86	1.15 (1.44)/0–4
4. Going shopping/ Ir de compras	0.86		0.74	0.84	1.59 (1.51)/0–4
5. Visiting friends/ Visitar a amigos		0.86	0.77	0.84	2.22 (1.61)/0–4
6. Participating in sports and recreation/ Deportes y entretenimiento		0.89	0.79	0.84	1.97 (1.66)/0–4
7. Going to work/ Ir a trabajar		0.52	0.49	0.87	0.82 (1.43)/0–4
8. Working on hobbies/ Practicar hobbies		0.96	0.83	0.83	2.22 (1.60)/0–4
9. Maintaining friendships/ Mantener amistades		0.71	0.69	0.85	1.69 (1.60)/0–4
Factor 1 ^a^	-	-	-	-	6 (4.68)/0–16
Factor 2 ^b^	-	-	-	-	8.91 (6.18)/0–20
Total ARS	-	-	-	-	14.91 (9.68)/0–36

Note: ^a^ instrumental activity restriction; ^b^ expressive activity restriction; r = Pearson correlation; All factor loadings and item-test correlations were significant (*p* < 0.01).

**Table 3:** Descriptive information on measures of leisure activities, behavioral activation, depressive and anxious symptoms, and perceived health, and their Pearson correlations with the ARS and ARS-OA, and their respective factors.

	Spanish Version of the ARS				ARS-OA			
	Total ARS	Factor 1 ^a^	Factor 2 ^b^	Mean (SD)/Range	Total ARS-OA	Factor 1 ^a^	Factor 2 ^b^	Mean (SD)/Range
Leisure Time Satisfaction scale (LTS)	−0.39 **	−0.30 **	−0.38 **	5.51 (2.62)/0–11	-	-	-	-
Activation subscale of the BADS-SF	-	-	-	-	−0.27 **	−0.25 **	−0.23 **	25.55 (4.54)/9–30
Center for Epidemiological Studies Scale (CES-D)	0.38 **	0.34 **	0.34 **	22.36 (11.87)/1–51	0.37 **	0.43 **	0.26 **	9.15 (7.41)/0–43
Tension-anxiety subscale of the POMS	0.26 **	0.23 **	0.23 **	19.70 (8.27)/1–36	-	-	-	-
Geriatric Anxiety Inventory (GAI)	-	-	-	-	0.30 **	0.36 **	0.21 **	6.21 (5.69)/0–22
Perceived health	−0.12	−0.15	−0.08	2.22 (0.91)/0–4	−0.52 **	−0.46 **	−0.48 **	2.61 (0.88)/1–4

Note: ^a^ instrumental activity restriction; ^b^ expressive activity restriction; ** *p* < 0.01; Perceived health: Higher scores indicate better perceived health.

**Table 4:** Factor loadings and descriptive analysis of the ARS-OA.

English/Spanish Items	Factor 1 ^a^	Factor 2 ^b^	Item-Test r	Cronbach’s α Without the Item	Mean (SD)/Range
1. Caring for self/ Cuidar de mí mismo	0.68		0.56	0.80	0.27 (0.67)/0–3
2. Caring for others/ Cuidar de otros	0.67		0.58	0.80	0.22 (0.66)/0–4
3. Doing household chores/ Hacer las tareas de la casa	0.83		0.69	0.78	0.41 (0.85)/0–4
4. Going shopping/ Ir de compras	0.82		0.63	0.79	0.28 (0.69)/0–4
5. Visiting friends/ Visitar a amigos		0.75	0.64	0.79	0.31 (0.78)/0–4
6. Participating in sports/ Realizar actividades deportivas		0.72	0.71	0.80	0.71 (1.14)/0–4
7. Participating in recreation activities/Realizar actividades de ocio		0.86	0.69	0.78	0.32 (0.79)/0–4
8. Working on hobbies/ Dedicarme a mis aficiones		0.85	0.71	0.78	0.32 (0.77)/0–4
9. Maintaining friendships/ Mantener mis amistades		0.65	0.47	0.81	0.13 (0.42)/0–2
Factor 1 ^a^	-	-	-	-	1.19 (2.07)/0–11
Factor 2 ^b^	-	-	-	-	1.79 (2.80)/0–15
Total ARS-OA	-	-	-	-	2.98 (4.38)/0–24

Note: ^a^ instrumental activity restriction; ^b^ expressive activity restriction; r = Pearson correlation; All factor loadings and item-test correlations were significant (*p* < 0.01).

## Data Availability

Data supporting the findings of this study are available upon reasonable request to the corresponding author.

## Supplementary Materials

The following supporting information can be downloaded at: https://www.mdpi.com/article/10.3390/geriatrics10010024/s1, Table S1: Items in English and Spanish from the ARS; Table S2: Items in English and Spanish from the ARS-OA.
