# Supplementary material for: Psychometric Properties of the Spanish Versions of the Activity Restriction Scale
Source: Geriatrics (Basel). 2025 Feb 10;10(1):24. doi: 10.3390/geriatrics10010024 (PMC11854992; doi:10.3390/geriatrics10010024)
Supplement: Supplementary file 1 [file geriatrics-10-00024-s001.zip › geriatrics-3401179-supplementary.pdf]

Supplementary Material

1. Activity Restriction Scale (ARS; [1]; in Spanish: “Escala de Restricción Conductual”)

1.1. Items

Table S1. Items in English and Spanish from the ARS.

English items [1]	Spanish items
1. Caring for self	1. Cuidado de sí mismo
2. Caring for others	2. Cuidado de otros
3. Doing household chores	3. Realizar tareas domésticas
4. Going shopping	4. Ir de compras
5. Visiting friends	5. Visitar a amigos
6. Participating in sports and recreation	6. Deportes y entretenimiento
7. Going to work	7. Ir a trabajar
8. Working on hobbies	8. Practicar hobbies
9. Maintaining friendships	9. Mantener amistades

1.2. Scoring instructions

Answers ranged from 0 (“never or seldom restricted” / “nunca o rara vez restringido”) to 4 (“greatly restricted” / “muy restringido”). The total ARS score is obtained by adding up the response options chosen for each of the items. Therefore, the total score can range from 0 to 36 points. Higher scores indicate higher levels of activity restriction.

1.3. References

1. Williamson, G.M.; Schulz, R. Activity restriction mediates the association between pain and depressed affect: A study of younger and older adult cancer patients. *Psychol. Aging.* **1995,** *10,* 369–378, https://doi.org/10.1037/0882-7974.10.3.369.

2. Older adults' version of the Activity Restriction Scale (ARS-OA; in Spanish "Escala de Restricción Conductual para Personas Mayores")

2.1. Items

Table S2. Items in English and Spanish from the ARS-OA.

English items	Spanish items
1. Caring for self	1. Cuidar de mí mismo
2. Caring for others	2. Cuidar de otros
3. Doing household chores	3. Hacer las tareas de la casa
4. Going shopping	4. Ir de compras
5. Visiting friends	5. Visitar a amigos
6. Participating in sports	6. Realizar actividades deportivas
7. Participating in recreation activities	7. Realizar actividades de ocio
8. Working on hobbies	8. Dedicarme a mis aficiones
9. Maintaining friendships	9. Mantener mis amistades

2.2. Scoring Instructions

Answers ranged from 0 ("never or seldom restricted" / "nunca o rara vez restringido") to 4 ("greatly restricted" / "muy restringido"). The total ARS-OA score is obtained by adding up the response options chosen for each of the items. Therefore, the total score can range from 0 to 36 points. Higher scores indicate higher levels of activity restriction.
